# BioSurfDB: knowledge and algorithms to support biosurfactants and biodegradation studies

**DOI:** 10.1093/database/bav033

**Published:** 2015-03-31

**Authors:** Jorge S. Oliveira, Wydemberg Araújo, Ana Isabela Lopes Sales, Alaine de Brito Guerra, Sinara Carla da Silva Araújo, Ana Tereza Ribeiro de Vasconcelos, Lucymara F. Agnez-Lima, Ana Teresa Freitas

**Affiliations:** ^1^INESC-ID/IST-Instituto de Engenharia de Sistemas e Computadores/Instituto Superior Técnico, Universidade de Lisboa, Rua Alves Redol 9, 1000-029 Lisboa, Portugal, ^2^Universidade Federal do Rio Grande do Norte, Natal, RN, Brazil and ^3^Laboratório Nacional de Computação Científica, Petrópolis, RJ, Brazil

## Abstract

Crude oil extraction, transportation and use provoke the contamination of countless ecosystems. Therefore, bioremediation through surfactants mobilization or biodegradation is an important subject, both economically and environmentally. Bioremediation research had a great boost with the recent advances in Metagenomics, as it enabled the sequencing of uncultured microorganisms providing new insights on surfactant-producing and/or oil-degrading bacteria. Many research studies are making available genomic data from unknown organisms obtained from metagenomics analysis of oil-contaminated environmental samples. These new datasets are presently demanding the development of new tools and data repositories tailored for the biological analysis in a context of bioremediation data analysis. This work presents BioSurfDB, www.biosurfdb.org, a curated relational information system integrating data from: (i) metagenomes; (ii) organisms; (iii) biodegradation relevant genes; proteins and their metabolic pathways; (iv) bioremediation experiments results, with specific pollutants treatment efficiencies by surfactant producing organisms; and (v) a biosurfactant-curated list, grouped by producing organism, surfactant name, class and reference. The main goal of this repository is to gather information on the characterization of biological compounds and mechanisms involved in biosurfactant production and/or biodegradation and make it available in a curated way and associated with a number of computational tools to support studies of genomic and metagenomic data.

Database URL: www.biosurfdb.org

## Background

Hydrocarbons are simple compounds of prime economic importance since they encompass the constituents of the major fuels (e.g. coal, oil, natural gas, etc.), as well as plastics, waxes, solvents and oils.

During hydrocarbon degradation, microorganisms generally produce adjuvant molecules called biosurfactants (1). Different microorganisms from several carbon sources can synthesize biosurfactants, being the synthesis influenced by the composition of the medium and by culture conditions. These amphipathic molecules can significantly reduce superficial tension in aqueous systems by accumulating in the interface and facilitating the emulsion of liquids with different polarities (2). The effects of biosurfactants on solubility, sorption and biodegradation of hydrophobic organic contaminants are well known (3), playing an important role in bioremediation of contaminated soil. Due to its properties, surfactants are widely applied in several industries, from laundry, to surface cleaning, additives for cement, cosmetics, pharmaceutics, agriculture, food industry and in oil industry (2).

The knowledge of bacterial and metabolic diversity is essential to understand the role of microbial communities in the different processes that occur in ecosystems. However, it is estimated that due to the difficulties of isolation and culture, a gene pool of 99% of microbial diversity is unknown (4). Recent advances in metagenomics have enabled the access to the genetic heritage of microbial species without the need for isolation and cultivation in the laboratory. With this methodology, it is possible to extract DNA from environmental samples such as soil or water which becomes available for various analyses, including large-scale sequencing (5). Presently, a wealth of information has been uncovered by metagenomics, such as: microbial diversity; vast swathes of uncharacterized metabolism; and increased complexity of biogeochemical pathways. Such data promises to provide knowledge about new enzymes and molecules with diverse applications.

Identifying and characterizing new genes involved in the degradation of hydrocarbons and production of surfactants, which have potential to develop a bioremediation strategy is thus promising and represents an important subject of research. For example, a large number of studies intend to evaluate the use of the identified genes and potential microbial consortia with large capacity of degradation for mature reservoirs recovery. These results may lead to the development of new biotechnological strategies and the creation of new industrial and biotechnological processes, important for preservation and environment planning.

Metagenomic data analysis is computationally demanding since it needs to deal with a mix of diverse genomes rather than DNA from a more homogeneous microbial population. One of the biggest challenges of computational metagenomics is making sense of the resulting data.

Metagenomic analysis software packages, like MG-RAST (6), MEGAN5 (7) and KRAKEN (8) normally include programs for taxonomic, functional and comparative analyses. Metagenomic datasets are crossed with huge databases, which combined with the constantly growing size of these datasets, produce large and complex outputs that usually take several days to be analysed.

The existence of accurate and efficient computational tools has shown an even greater impact on metagenomic studies when compared with traditional genomic projects, due not only to the large amount of data, but also to the new complexity introduced by this data. One of the first steps of the analysis of a DNA sequencing dataset is genome assembly. Unfortunately, due to the high number of species under analysis and to the short length of sequencing reads obtained from next generation sequencers, the genome assembly goal is too difficult if not impossible to attain for samples from many microbial environments. As a result, metagenomic datasets are often subject to further analysis as a collection of short reads. Since one of the primary goals of metagenomic projects is to characterize the organisms present in an environmental sample, a number of tools have been developed to perform similarity-based or phylogeny-based searches of metagenomic sequences on databases of known genes or proteins. For a number of problems, the existence of curated databases including only contextual data, useful for sequence and functional annotation, can play an important roll for denoising data analysis results and highlight small but important signals in the data. Biosurfactants and biodegradation studies are examples of problems which may benefit from the development of specialized information systems including data about organisms producing biosurfactants; biodegradation relevant genes, proteins and their metabolic pathways; bioremediation experiments results, with specific pollutants treatment efficiencies by surfactant producing organisms, etc. These systems should also provide computational tools for the analysis of new genomic and metagenomic datasets in a fast and accurate way. Databases like the carbohydrate active enzymes (CAZy) (9), University of Minnesota Biocatalysis/Biodegradation Database (10) or OxDBase (11) are focused on presenting data without providing tools for the comparison of the database content with the user own data. They do not provide interfaces or tools for an interactive data analysis. Databases like NCBI (12) or UniProt (13) are to generic, making references to ‘surfactants’, ‘biodegradation’ or ‘bioremediation’ data from very distinct sources and distinct research fields. The large spectrum of the data available makes the data integration and curation of new short read datasets harder and time consuming, forcing the need for endless filtering and post-processing data procedures.

BioSurfDB was built as an answer to the aforementioned challenges. Not only it contains important data to support biosurfactants and/or biodegradation studies, but also combines a set of tailored tools to enable, in an efficient way, specific metagenomic analysis. The main goal of this new tool is to support (i) the identification of patterns of taxonomic and functional diversity of microbial communities and (ii) the identification of novel genes involved in the degradation of hydrocarbons and surfactants production which have potential for the development of bioremediation strategies.

### BioSurfDB data description

The BioSurfDB system database model was designed to enable the modeling of the main concepts and relations in the surfactant production and biodegradation domains. Supplementary Figure S1 presents a detailed version of the developed relational database model. This model was implemented in mySQL. *Perl* scripts have been created to support GenBank (14) data filtering and uploading. All data is represented using standard file formats, like FASTA (15) for the nucleic or proteic sequences, KEGG (16) for pathways and PUBMED for article references. URI links have been included to connect this repository data to external sources, aiming to support a linked data policy.

In this first version, the system makes available data about 3736 genes, 3430 proteins, 1077 organisms, 58 pathways, 47 detailed bioremediation experiments, with specific pollutants treatment efficiencies by surfactant producing organisms and a 96 biosurfactants-curated list, grouped by producing organism, surfactant name, class and references.

To the best of our knowledge, this domain-specific database includes the most updated dataset on biosurfactants-producing bacteria, including 274 associations between organisms, genes, proteins and metabolic pathways. Biodegradation data represents an important fraction of the database content. Although this type of data can also be found in existent databases, e.g. University of Minnesota Biocatalysis/Biodegradation Database (10), it is made available in this systems associated with a number of computational tools that helps researchers improve their data analysis.

## Data exploitation and data services

The BioSurfDB website makes available a number of computational tools to support the exploitation of DNA and protein sequence datasets as it is exemplified in the Website Tutorial, biosurfdb.org/tutorial.php. A set of queries has been preprogrammed and Clustal (17) and BLAST (18) sequence aligners have been made available in order to help users retrieve biological information and predict the existence of conserved genes and proteins among different microorganisms sequence sets or metagenomes. The seven examples below detail some of the most important actions that can be performed using this new system. All these examples are detailed in the BioSurfDB Website Tutorial.

### Example 1: Annotate your sequences using BLAST

Sequence annotation is of the utmost importance as a first step of sequence analysis. The BioSurfDB system makes available all types of protein and nucleotide BLAST algorithms and BLAST databases including all the protein and genes sequences available in the system. It is possible to BLAST one or multiple sequences against a selected database in the *BLAST Sequence* Menu. *Expect threshold* and *word size* refinements are also available to enhance the search.

Standard output formats have been made available including an HTML output generated with the Mview (19), a software that helps the visualization of the alignment in the browser by coloring identical sequences and showing the percentage identity between the sequences. This visualization option is critical for biologists that need to amplify/clone the sequences under analysis since it supports the definition of primers designs.

### Example 2: Discover taxonomy and function in your sequences

One of the output options of BLAST that deserves be highlighted is the Abundance Analysis. By selecting this option it is possible to characterize the dataset considering the following features: (i) the organism abundance and diversity; (ii) gene or protein sequence counts; and (iii) the distribution of the sequences per metabolic pathway. This characterization presents a number of histograms and can be performed for input datasets including genomes or metagenomes.

The Abundance Analysis option activates a postprocessing pipeline that: (i) Selects the best hit of each read alignment query, based on the best score. This selection guaranties that each read corresponds to only one hit—the best hit, preventing that one read that aligns to multiple places can introduce noise on the final result; (ii) Identifies the organism’s name, gene or protein and metabolic pathway where the read belongs to; (iii) Creates histograms characterizing the input dataset for the features previously described.

**Figure 1.**
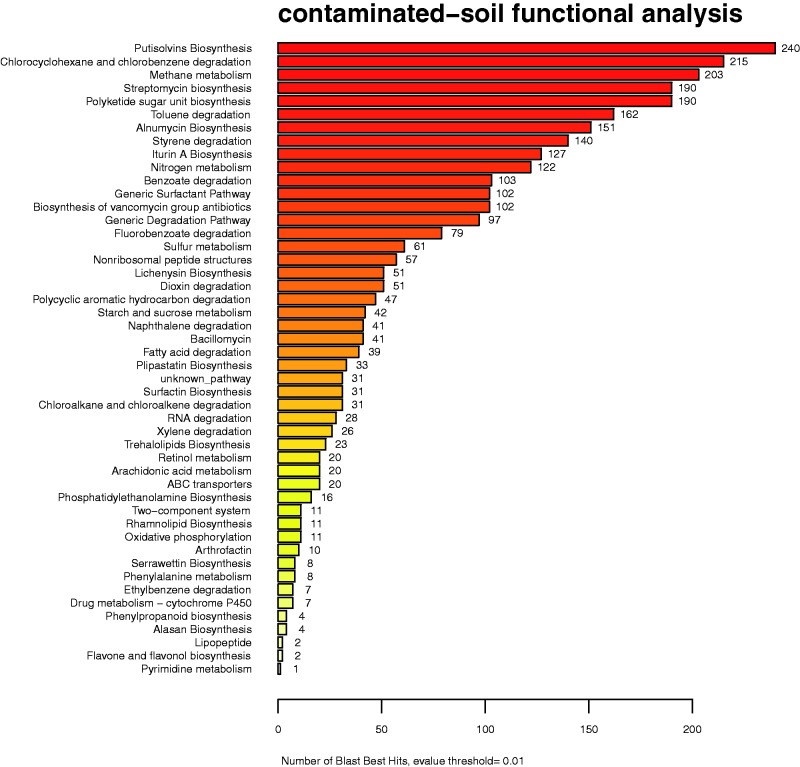
Abundance analysis of an oil-contaminated soil sample. An important number of proteins involved in the synthesis of biosurfactants and alkane degradation have been identified.

Figure 1 presents the Abundance Analysis of a metagenome dataset including a microbial consortium isolated from oil-contaminated soil. With this analysis it was possible to explicitly identify several biosurfactant synthesis pathways, enabling the identification of an important number of proteins involved in the synthesis of biosurfactants and alkane degradation.

### Example 3: Explore the BioSufDB data on surfactant production and biodegradation

To better explore the information on Surfactant production and Biodegradation, a tabular representation containing the association between organisms, genes, proteins and metabolic pathways is presented though the *List Of* menu. Hyperlinks have been used to quickly access the data details. For the gene and protein list, the *GI field* is presented as a link to the correspondent sequences (nucleic or proteic). In the details page, the *GI attribute* is a link to external sources of information (NCBI, UniProt, etc).

### Example 4: Find gene or protein homologs using Clustal multiple alignment

In order to help users to understand gene or protein homology across species or strains, an option was created to enable the automatic comparison between genes or proteins sequences presented at the ‘Organism to Pathway’ association table, obtained though the *List Of* menu. In this table, two or more genes can be selected and aligned using the Clustal algorithm. The output is an HTML page generated with the aforementioned Mview visualization tool.

### Example 5: Explore networks

As explained in the example 3, a tabular representation containing the association between organisms, genes, proteins and metabolic pathways can be visualized through the ‘*List Of’* menu.

Organizing this tabular representation in a simple network can help biologists to better answer questions such as: ‘What is the complete set of genes and proteins in a species?’, ‘What is the complete set of organisms producing a certain protein?’ or ‘Which organisms participate in a specific hydrocarbon degrading pathway, and which genes do they contribute with?’*.* To help answering these types of questions a visualization tool was made available in the Organism to Pathway list menu. With this tool it is possible to select two or more table entries and ask for a network to be designed. The selected information is used by a graph generation program (Graphviz) that groups identical columns like, organisms, genes, proteins, etc.

**Figure 2.**
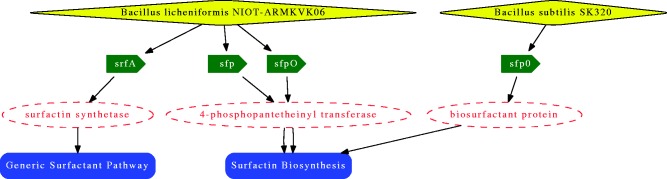
Surfactin biosurfactant biosynthesis pathway. From the top to bottom: Organisms (yellow diamond), genes (green arrow), proteins (red dashed circle) and pathway (blue ribbon).

Figure 2 shows the network obtained when selecting the surfactin (biosurfactant) biosynthesis pathway from the tabular representation containing the association between organisms, genes, proteins and metabolic pathways. This tabular representation in obtained through ‘List Of’ menu.

This network can help answering the following question: ‘Which organisms, genes and proteins are involved in the surfactin (biosurfactant) biosynthesis pathway?’. As it shows this surfactant is produced by Bacillus species and four genes and three proteins are involved.

### Example 6: Explore published bioremediation studies

A large number of bioremediation experiments (20–22) have been made publicly available throughout the years; however, the lack of a database to store and view that data hampers its use. To support answering questions like ‘Which organisms has already been characterized in the context of bioremediation of contaminated soils?’ or ‘Which class of surfactants is more efficient for the elimination of a certain pollutant?’, BioSurfDB provides a library containing the most relevant details on a number of publicly available bioremediation experiments. This library presents data about organisms, surfactants, pollutants and bibliographic references, providing a data integration environment to end-users. This effort to bring together data already available, as a result of a number of important research studies, should be seen as the first step of a more ambitious project that has the objective to build a new virtual research environment for data sharing and reuse. This library is available through the ‘List Of’ menu, in an entry named Documented Bioremediation Results. This data, that is representative of environments that need bioremediation interventions, can also be used to characterize new datasets and to compare different ecosystems.

### Example 7: Analyze your own metagenome

As already stated, one of the main purposes of this new system is to provide computational tools to support metagenomes data analysis. In this first version, the system only allows the automatic upload of medium sized datasets, including data from bacterial genomes or just a set of DNA or protein sequence to be used on the different comparisons. However, metagenomic datasets, usually very large datasets, can also be explored within BioSurfDB. Due to its sizes, a mechanism for the automatic upload from the Website is still under evaluation. Presently, the users can contact the BioSurdDB system Admin, thought the *Contact Form,* in order to ask for the upload of their own data. Once uploaded, the data will be made available as a library of sequences that can be used to BLAST against other databases available at the system.

BioSurfDB already makes available, for sequence comparison, a public metagenome from GENOREM (23) a project focused on oil-contaminated soils.

## Discussion

As exemplified in the Data Exploitation and Data Services Section, BioSurfDB brings together data and computational tools that differentiate it positively from other databases on bioremediation domain.

Table 1 presents a comparison between a number of existent data resources, focusing on features like the existence of data analysis services and data statistics.

**Table 1.** Biosurfactants and biodegradation databases comparison

	BioSurfDB	UM-BBD (10)	OxDBase (11)	CAZy (9)
Focus	Biosurfactants and biodegradation	Biocatalysis and biodegradation	Biodegradative oxygenases	Carbohydrate active enzymes
Search and view data	Yes	Yes	No	Yes
BLAST service	Yes	No	No	No
Sequence and analysis service	Yes	No	No	No
Number of species^a^	1077	248	NA	1436
Number of proteins	3430	993	235	340 000
Number of pathways	58	219	NA	NA
Number of genes	3736	NA	NA	NA

^a^Not accounting for strains.

The presented databases have been selected since they make available important genomic data on biosurfactant production and biodegradation. OxDBase is a database focused on a specific group of enzymes—Oxygenases, while CAZy database covers all groups. UM-BBD and BioSurfDB information systems present data about many biological entities and are not focused on enzymes uniquely. BioSurfDB is unique regarding the BioSurfactants domain.

Except for OxDBase, that only allows a simple search operation, all the other databases provide a list visualization of all the data available.

As for the BLAST and Sequence analysis services, BioSurfDB is the only system that provides those services, associated with the fact that is the only database that effectively stores sequence (nucleic or proteic) information.

By bringing together these data analysis services, BioSurfDB supports in a very efficient way several research tasks in the context of the analysis of a genomic dataset from a bioremediation study. For instance, to reproduce with the publicly available data and computational tools the *BioSurfDB BLAST + Functional Analysis* service, one would have to perform the following tasks: (i) query a publicly generic genomic data database for sequence similarity; (ii) follow the Genbank or UniProt external link to validate the importance of each result, in the context of bioremediation; (iii) Upload the selected reads, from the dataset under analysis, into a taxonomic, functional and comparative analyses software, like MEGAN and finally (iv) Generate the taxonomic and functional graphics. This normally used selection process is time consuming and can loose weak but important signals present in the original dataset. BioSurfDB provides an integrated environment to improve this type of analysis procedures.

Regarding the data statistics on the number of species, enzymes and pathways, the CAZy database is an interesting resource as it contains a very wide spectrum of species and enzymes. However, it does not contains pathways like UM-BBD and BioSurfDB and Genes like BioSurfDB.

## Future work

The main goal of this work is to create a domain-specific system, in the field of Bioremediation, in order to enhance the view of the existent relations between metagenomes, organisms, genes, proteins and degradation pathways. The system is also a contribution to the linked data world movement though the use of links (URIs) to connect the existence data with data and concepts on other well-known repositories. One example is the connection of pathways to functional classification databases as KEGG (16), COG (24) and SEED (25). By being a domain-specific system, it enables users to focus on the characterization of their datasets considering only information on organisms of interest.

In its actual state, the BioSurfDB system already proved its importance in the analysis of microbial consortium metagenomes, sequenced with IonTorrent Ion PGM and isolated from oil-contaminated soil samples, by suggesting the organism, gene, protein and pathway relative abundances and consequently assisting the decision of choosing the best microbial consortium for the task of bioremediation.

To keep improving the importance and usefulness of this new resource, new queries and algorithms are already planned to be developed. New actions will consider the development of a pipeline to enable the identification of ORFs in metagenomic datasets. The pipeline will include programs for the reads quality control; contigs builder; and Short-Read Sequence Aligners, like TAPyR (26) or BWA (27), to map reads against reference genomes. It is also planed to support the development of a virtual research environment to increase data sharing and reuse. Cloud services will also be considered to support metagenomes datasets uploads.

## Supplementary Data

Supplementary data are available at *Database* Online.

## Funding

This work was supported by national funds through Fundação para a Ciência e a Tecnologia (FCT) with reference UID/CEC/50021/2013. FAPERJ Bolsa Doutorado-Sanduiche Reverso (E-26/101.230/2014 and CNPq e CAPES).

There is no open access publication policy defined. All funds have been acknowledged.

*Conflict of interest*. None declared.

## Supplementary Material

Supplementary Data

## References

[1] Banat I.M. (1995) Biosurfactants production and possible uses in microbial enhanced oil recovery and oil pollution remediation: A review. Bioresour. Technol., 51, 1–12.

[2] Marchant R. Banat I.M. (2012) Microbial biosurfactants: challenges and opportunities for future exploitation. Trends Biotechnol., 30*,* 558–565. 22901730 10.1016/j.tibtech.2012.07.003

[3] Bustamante M. Durán N. Diez M.C. (2012) Biosurfactants are useful tools for the bioremediation of contaminated soil: a review. J. Soil Sci. Plant Nutr., 12, 667–687.

[4] Handelsman J. Rondon M.R. Brady S.F. (1998) Molecular biological access to the chemistry of unknown soil microbes: a new frontier for natural products. Chem. Biol., 5, R245–R249. 9818143 10.1016/s1074-5521(98)90108-9

[5] Handelsman J. (2004) Metagenomics: application of genomics to uncultured microorganisms. Microbiol. Mol. Biol. Rev., 68, 669–685. 10.1128/MMBR.68.4.669-685.2004 PMC539003 15590779

[6] Meyer F. Paarmann D. D'Souza M. (2008) The metagenomics RAST server – a public resource for the automatic phylogenetic and functional analysis of metagenomes. BMC Bioinform., 9, 1–8. 10.1186/1471-2105-9-386 PMC2563014 18803844

[7] Huson D.H. Mitra S. Ruscheweyh H. (2011) Integrative analysis of environmental sequences using MEGAN4. Genome Res., 21, 1552–1560. 21690186 10.1101/gr.120618.111 PMC3166839

[8] Wood D.E. Salzberg S.L. (2014) Kraken: ultrafast metagenomic sequence classification using exact alignments. Genome Biol., 15, R46. 24580807 10.1186/gb-2014-15-3-r46 PMC4053813

[9] Lombard V. Golaconda R.H. Drula E. (2014) The Carbohydrate-active enzymes database (CAZy) in 2013. Nucleic Acids Res., 42, D490–D495. 24270786 10.1093/nar/gkt1178 PMC3965031

[10] Lynda B.M.E. Lawrence PW. (2012) Use of the University of Minnesota Biocatalysis/Biodegradation Database for study of microbial degradation. Microb. Inform. Exp., 2, 1–10. 22587916 10.1186/2042-5783-2-1 PMC3351732

[11] Arora P.K. Kumar M. Chauhan A. (2009) OxDBase: a database of oxygenases involved in biodegradation. BMC Res. Notes, 2, 67. 19405962 10.1186/1756-0500-2-67 PMC2683861

[12] Geer L.Y. Marchler-Bauer A. Geer R.C. (2010) The NCBI BioSystems database. Nucleic Acids Res., 38. 10.1093/nar/gkp858 PMC2808896 19854944

[13] UniProt: the Universal Protein Resource (www.uniprot.org).

[14] Benson D.A. Cavanaugh M. Clark K. (2013) GenBank. Nucleic Acids Res., 41, 36–42. 10.1093/nar/gks1195 PMC3531190 23193287

[15] Pearson W.R. (1990) Rapid and sensitive sequence comparison with FASTP and FASTA. Methods Enzymol., 183, 63–98. 2156132 10.1016/0076-6879(90)83007-v

[16] Kanehisa M. Goto S. (2000) KEGG: Kyoto encyclopedia of genes and genomes. Nucleic Acids Res., 28, 27–30. 10592173 10.1093/nar/28.1.27 PMC102409

[17] Sievers F. Higgins D.G. (2014) Clustal omega, accurate alignment of very large numbers of sequences. Methods Mol. Biol., 1079, 105–116. 24170397 10.1007/978-1-62703-646-7_6

[18] Altschul S.F. Gish W. Miller W. (1990) Basic local alignment search tool. J. Mol. Biol., 215, 403–410. 2231712 10.1016/S0022-2836(05)80360-2

[19] Brown N.P. Leroy C. Sander C. (1998) MView: a web-compatible database search or multiple alignment viewer. Bioinformatics, 14, 380–381. 9632837 10.1093/bioinformatics/14.4.380

[20] Bondarenko O. Rahman P.K. Rahman T.J. (2010) Effects of rhamnolipids from Pseudomonas aeruginosa DS10-129 on luminescent bacteria: toxicity and modulation of cadmium bioavailability. Microb. Ecol., 59, 588–600. 20082071 10.1007/s00248-009-9626-5

[21] Franzetti A. Gandolfi I. Bestetti G. (2010) Production and applications of trehalose lipid biosurfactants. Eur. J. Lipid Sci. Technol. Microb. Biosurf., 112, 617–627.

[22] Ławniczak L. Marecik R. Chrzanowski L. (2013) Contributions of biosurfactants to natural or induced bioremediation. Appl. Microbiol. Biotechnol., 97, 2327–2339. 23400445 10.1007/s00253-013-4740-1 PMC3585901

[23] Burger G. Courchesne F. Greer C. (2012) Genorem: improving bioremediation of polluted soils through environmental genomics. Environ. Eng. Manag. J., 11, No. 3, Supplement, S22.

[24] Tatusov R.L. Galperin M.Y. Natale D.A. (2000) The COG database: a tool for genome-scale analysis of protein functions and evolution. Nucleic Acids Res., 28, 33–36. 10592175 10.1093/nar/28.1.33 PMC102395

[25] Overbeek R. Begley T. Butler R.M.*.* (2005) The subsystems approach to genome annotation and its use in the project to annotate 1000 genomes. Nucleic Acids Res., 33, 5691–702. 16214803 10.1093/nar/gki866 PMC1251668

[26] Fernandes F. da Fonseca P.G. Russo L.M. (2011) Efficient alignment of pyrosequencing reads for re-sequencing applications. BMC Bioinform., 12, 163. 10.1186/1471-2105-12-163 PMC3118166 21672185

[27] Li H. Durbin R. (2009) Fast and accurate short read alignment with Burrows–Wheeler transform. Bioinformatics, 25, 1754–176. 19451168 10.1093/bioinformatics/btp324 PMC2705234

