# Supplementary material for: BioSurfDB: knowledge and algorithms to support biosurfactants and biodegradation studies
Source: Database (Oxford). 2015 Mar 31;2015:bav033. doi: 10.1093/database/bav033 (PMC4381105; doi:10.1093/database/bav033)
Supplement: Supplementary Data [file supp_2015_bav033_index.html]

Supplementary Data 

# BioSurfDB: knowledge and algorithms to support biosurfactants and biodegradation studies

## Supplementary Data

files

**Files in this Data Supplement:**

- Supplementary Data - zip file
